# The plans of Ernesto Ottoni to Combat the “affliction of lepers:” lazarettos, treatments, and endeavors to tackle leprosy in nineteenth century São Paulo

**DOI:** 10.1590/S0104-59702023000100044

**Published:** 2023-09-04

**Authors:** Jean Luiz Neves Abreu, Lucas Samuel Quadros

**Affiliations:** i Professor, Universidade Federal de Uberlândia.Uberlândia – MG – Brasil jeanluiz.na@gmail.com; ii Professor, Secretaria de Educação do Estado de Minas Gerais. Paraopeba – MG – Brasil lucassquadros@yahoo.com.br

**Keywords:** Ernesto Benedicto Ottoni (1816-1881, Leprosy, Lazaretto, Lepers, São Paulo, Ernesto Benedicto Ottoni (1816-1881, Lepra, Lazaretos, Morféticos, São Paulo

## Abstract

This article presents the plans for a lazaretto in the city of São Paulo in the mid-nineteenth century. It consists of the transcription and analysis of an opinion prepared by the physician Ernesto Benedicto Ottoni, addressed to the president of the province of São Paulo. The article includes an analysis of the plans for the building, bearing in mind the prevailing miasma theory; the contemporary conceptions of leprosy treatment, especially beliefs regarding the transmissibility of the disease; and the physician’s idealization of the routines for the treatment, work, leisure, and recovery of patients.

## Resumo

O trabalho a seguir objetiva apresentar o projeto de estabelecimento de um lazareto para morféticos na cidade de São Paulo em meados do século XIX. Trata-se da transcrição e análise de um parecer elaborado pelo médico Ernesto Benedicto Ottoni e endereçado ao presidente da província de São Paulo. Analisamos os planos da construção do prédio, principalmente levando em consideração a teoria dos miasmas; concepções sobre as terapêuticas da lepra na época, sobretudo quanto à transmissibilidade da doença; e, por fim, a idealização do médico acerca das rotinas de tratamento, trabalho, lazer e recuperação dos doentes.

O documento que apresentamos ao leitor é a transcrição de um parecer referente às necessidades de construção de um local para os doentes "morféticos"[1] em São Paulo. Trata-se de um relatório elaborado pelo médico Ernesto Benedicto Ottoni, endereçado, na forma de ofício, ao presidente da província de São Paulo, José Antonio Saraiva, em 1855, e que tratava do projeto de construção de um lazareto para os doentes de lepra.

O manuscrito encontra-se digitalizado e disponibilizado no *site* do Arquivo do Estado de São Paulo e integra o acervo do projeto "Memórias Paulistanas: preservação e disponibilização de ofícios diversos de São Paulo (1822-1919)", cujo banco de dados possibilita o acesso às correspondências recebidas pelo governo de São Paulo entre 1822 e 1921 (http://www.arquivoestado.sp.gov.br/web/digitalizado/textual/oficios_diversos).

Toda a análise foi feita a partir de transcrição integral do ofício, que desde sua leitura primária mostrou-se rico em detalhes argumentativos, citações e proposições, justificando seu exame completo. Apesar de não se tratar de documento inédito, tendo sido brevemente mencionado no *Tratado de leprologia*, de Flávio Maurano (1950, p.85), sua relevância como fonte para o estudo da lepra e sua divulgação justificam a transcrição e divulgação. Nesse sentido, ressalta-se o papel que a pesquisa documental assume para a história das ciências da saúde, permitindo reconstituir saberes e práticas médicas do passado. Do ponto de vista teórico-metodológico, para a análise da fonte, além de observar a dinâmica internalista da produção de conhecimento sobre a lepra, atentou-se igualmente para as condições políticas e sociais daquele contexto. A esse respeito, Maria Rachel Fróes da Fonseca (2002, p.276-277) observou que "não se deve adotar uma visão descontextualizada da atividade científica, ou centrar-se unicamente na dinâmica internalista deste processo, ou seja, no estudo da estrutura conceitual e lógica do conhecimento científico". Além disso, as análises devem levar em conta os atores, os espaços institucionais onde atuam e os espaços de representação (associações, sociedades e periódicos).

Atendendo a esses pressupostos, cabe aqui contemplar, ainda que de forma resumida, a trajetória de Ernesto Ottoni. As referências de que dispomos são escassas na historiografia. Seu nome, entretanto, é normalmente referenciado em obras dedicadas às efemérides. Tais obras permitem localizar algumas informações genéricas sobre sua trajetória profissional, como, por exemplo, sua atuação como médico na província de São Paulo e homem de ciência dedicado aos estudos sobre o cólera (Veiga, 1897, p.440-441; Senna, 1905, p.202).

A partir das informações disponíveis nessas obras e em textos publicados na imprensa, é possível reconstituir alguns aspectos da vida de Ernesto Ottoni. Médico natural do Serro (MG), era filho de Jorge Ottoni e Rosália de Souza Maia, importante família da região. Um dos irmãos de Ernesto era Teófilo Ottoni, personagem que se destacou na vida política do Império, como deputado e fundador da Companhia do Mucuri (Duarte, 2002). Ernesto Ottoni, por sua vez, optou pela carreira de medicina, indo para a Corte provavelmente em 1835. Durante o curso, morou na residência de seu outro irmão, Cristiano Ottoni, que, além de engenheiro, foi senador e capitão-tenente da Marinha. Tendo concluído o curso de medicina em 1841, tudo indica que ele permaneceu no Rio de Janeiro até 1847, quando se dirigiu para a região do Mucuri, por um breve espaço de tempo (Navegação..., 1847, p.476).

Encontramos referências da presença de Ernesto Ottoni na sede da província de São Paulo já em 1850, onde permaneceu até 1856, quando retornou, por alguns anos, à região do Mucuri (MG), onde participou da empresa de colonização da região idealizada pelo irmão, Teófilo Ottoni. O médico assumiu um papel de destaque na Companhia, tornando-se subdelegado da Filadélfia, tendo por função arbitrar sobre diversas questões da empresa, além de prestar assistência médica aos colonos da região (Relatório..., 1 jul. 1860, p.2).[2] Ernesto Ottoni voltou para o Rio de Janeiro no início da década de 1860, sendo contratado pelo quartel-general da Marinha, em 1865, para servir no hospital da Marinha da Corte, como segundo cirurgião da Comissão (Notícias diversas, 26 nov. 1865, p.1). O médico morreu em 1881, segundo a autobiografia de seu irmão Cristiano, como "um bom velho que vivia já de anos em estado de profunda anemia" (Ottoni, 2014, p.226).

Não obstante a carreira de Ernesto Ottoni merecer estudo mais aprofundado, o propósito deste artigo é destacar o período em que ele esteve na cidade de São Paulo, entre 1850 e 1860. O período em que o médico esteve na província seria marcado pela preocupação com questões de higiene pública, a situação das prisões, do matadouro e dos cemitérios (Mantovani, 2022). No século XIX, havia doenças endêmicas graves em todo o território habitado de São Paulo, sendo algumas vezes tratadas como epidemias, normalmente quando atingiam os estamentos superiores da sociedade. Em meados da década de 1850, o cólera e a febre amarela eram epidemias que afetavam a província e a capital paulista (Mantovani, Marques, 2019, p.422-433).

Ernesto Ottoni desempenhou papel importante nas medidas sanitárias tomadas em São Paulo. Além das atividades clínicas e de serviços de corpos de delito que prestou à Câmara Municipal, ele integrou diversas comissões de salubridade a serviço do poder camarário e da província, entre 1850 e 1856. Essas comissões, compostas por médicos, engenheiros e demais autoridades, eram destinadas a tratar das questões de salubridade, como, por exemplo, a construção de matadouros e cemitérios fora da cidade, exame da qualidade da água, entre outras. Eram formadas por iniciativa da administração pública, que buscava contornar os problemas sanitários que atingiam a cidade e a província de São Paulo (Mantovani, 2015, p.122-157). Além de participar de comissões destinadas a questões de salubridade pública, Ottoni também integrou outras, voltadas para problemas relativos às epidemias do século XIX, a exemplo da febre amarela e do cólera (Atas..., 1939a, p.234, 1939b, p.82).

## A lepra em São Paulo e o projeto do lazareto

No período em que Ernesto Ottoni elaborou seu parecer, a lepra não era uma doença desconhecida na região. Em artigo que trata da expansão da hanseníase, Letícia Maria Eidt (maio-ago. 2004, p.81) observa que, já no século XVIII, há referências ao aparecimento da hanseníase, em "carta enviada ao Conde de Oyeiras, em 1765, em que o governador se refere ao perigo que corria a capitania em decorrência da doença". Em 1822, houve um primeiro censo sobre o número de casos da doença, coordenado pelo então governador interino das Armas da província de São Paulo, José Arouche de Toledo Rendon. De acordo com os dados levantados, havia um considerável número de pessoas acometidas pela enfermidade, em diversas cidades da província (Mantovani, Marques, 2019, p.426). "Outros censos foram realizados depois, notando-se sempre um aumento do número de pacientes, o que acompanhava a marcha do progresso do Estado" (Eidt, maio-ago. 2004, p.81). Adolpho Lutz, que se dedicaria ao estudo da doença até o final de sua vida, estimava que em Limeira, onde era clínico, chegou a tratar, no final de 1888, de 200 a 250 pacientes, sendo a cidade um dos locais mais afetados do país segundo o médico (Benchimol, Sá, 2004, p.33).

Em razão dos casos de lepra em São Paulo, fazia-se necessário tomar medidas visando enfrentar a doença e sanar a questão dos morféticos que andavam pelas ruas. Nesse contexto foi produzido o documento aqui transcrito. No discurso apresentado na Assembleia Legislativa, em 15 de fevereiro de 1855, o presidente da província, José Antonio Saraiva, considerava que o estado sanitário de São Paulo era satisfatório, embora se tivesse a "morfeia" como a doença preocupante da região. Diante disso, informava que havia uma comissão composta por Ernesto Ottoni, Antônio Roberto de Almeida e o major Luiz José Monteiro, e incumbida de apresentar os parâmetros para a confecção do regulamento policial e sanitário. Apesar de ser fruto do trabalho de toda a comissão, José Antonio Saraiva se referia somente a Ernesto Ottoni como autor do documento (Discurso..., 17 fev. 1855, p.1).[3] Já em 8 de fevereiro do mesmo ano, Ottoni relatava que a comissão sugeria que o regulamento policial e sanitário deveria ser confeccionado, de fato, somente após a construção de um grande lazareto, capaz de recolher os "morféticos".

O relatório de Ernesto Ottoni vinha ao encontro das propostas que visavam ao isolamento dos doentes para o tratamento da lepra. O texto apontava a necessidade de se construir um prédio, indicando a Chácara de Sant'Anna como o melhor local para sua edificação. Um dos primeiros aspectos destacados era quanto às condições de salubridade do lugar onde o lazareto deveria ser levantado. Considerava que a chácara era um lugar adequado, por se situar em terreno distante da cidade e em exposição favorável aos "raios do sol e os ventos, que renovam constantemente a atmosfera" (Ottoni, 8 jan. 1855, fl.3).

A designação "lazareto" remetia a uma das denominações da doença no século XIX, também conhecida como "mal de lázaro", sendo os leprosos comumente chamados de "lázaros". O estabelecimento de espaços dessa natureza consistia em uma das principais medidas tomadas em relação aos adoecidos desde a Idade Média. Segundo George Rosen (1994, p.60-61), pelo temor que causava e pela necessidade de controlar a propagação, a principal "ação de saúde pública" era o isolamento das pessoas acometidas pela doença, que por muito tempo foi considerada contagiosa. A "possibilidade de que a enfermidade afetasse os demais, não apenas lhes tirando a saúde, mas a estética e a beleza, acabou tornando legítimo o uso do afastamento institucionalizado" (Mattos, Fornazari, 2005, p.50).

No Brasil, os indícios mais antigos de iniciativas de isolamento datam de 1640, quando foi criado, em Salvador, o Campo dos Lázaros. No Rio de Janeiro, diante das pressões para remover as vítimas das ruas da cidade, foi fundado, em 1763, no bairro de São Cristóvão, o hospital conhecido como Hospital dos Lázaros, administrado pela Irmandade do Santíssimo Sacramento da Candelária (Smith III, 2003). No século XIX, esse estabelecimento se tornou um "importante centro de experimento em torno da terapêutica e etiologia da lepra" (Cabral, 2006b, p.42).

A construção de locais destinados a acolher os doentes de lepra foi comum no período. Em muitas cidades foram construídos abrigos. Entretanto, nem sempre havia a presença de médicos, sendo a assistência fornecida por religiosos e leigos. A precariedade de hospitais e instituições dedicadas ao tratamento da lepra no país tornou-se motivo de diversas críticas, fosse pela estrutura de assistência ou pelos métodos de cura empregados (Silva, 2017, p.227).

Em São Paulo, ainda no século XVIII, o aumento dos casos de lepra fez com que o governador da capitania, Morgado de Mateus, além das preces públicas, ordenasse erigir um hospital, por volta de 1765. Mas a construção do estabelecimento acabou acontecendo somente no século seguinte,[4] com o Hospital dos Lázaros de São Paulo, mantido pela Santa Casa de Misericórdia, com o auxílio do governo (Maurano, 1950, p.102; Maciel, Ferreira, 2014, p.22).

Havia, entretanto, falta de recursos no hospital, e o atendimento médico aos doentes era tido como péssimo. O espaço era reduzido, o que permitia o atendimento a poucas pessoas, servindo a instituição quase que somente como forma de isolar os enfermos (Jorge, 2006, p.54-57). Tal situação não era alheia a Ernesto Ottoni. Seu parecer relatava que os "morféticos" no Hospital dos Lázaros viviam "em completo abandono, e morrem à míngua sem receber o menor socorro da Medicina" (Ottoni, 8 jan. 1855, fl.17). Havia, dessa forma, a necessidade de construção de um lazareto capaz de socorrer e abrigar os doentes que abundavam em diversos pontos da província.

Previa-se o estabelecimento de um edifício capaz de abrigar duzentos doentes, de ambos os sexos. Para sua construção, seria importante observar à risca as leis da salubridade, higiene e estética da arquitetura. Nesse sentido, o médico apontava a necessidade de renovação do ar por janelas largas, evitando que os miasmas se acumulassem na parte superior do prédio. Também pedia a instalação de ventiladores, objetivando renovar o ar na parte inferior do edifício.

Os aspectos a ser observados na edificação iam ao encontro da literatura médica da época, que preconizava a renovação do ar como forma de impedir a ação dos miasmas, vistos como um dos principais fatores predisponentes de diversas enfermidades no século XIX. O mesmo receio se estendia a outros locais, como cemitérios, prisões e hospitais, os focos da vigilância olfativa (Corbin, 1987). Conforme já amplamente discutido pela historiografia, no Brasil, a ação exercida pelos miasmas morbíficos era considerada uma das principais causas de inúmeras enfermidades, a exemplo do cólera e da febre amarela (Santos, 2016; Kury, 2007, p.160-162; Chalhoub, 1996, p.64-65).

Além de apontar os elementos higiênicos a seguir na construção do edifício, Ottoni também propunha que, no terreno da chácara, fosse destinado um espaço para a plantação de hortas, jardins e pomares para recreação dos internos. Também se cultivariam "ervas frescas e antiescorbúticos" para uso dos doentes e, em maior escala, as plantas de "raízes tuberosas", como o inhame, considerado alimento "preservativo da morfeia" (Ottoni, 8 jan. 1855, fl.7).

A terapêutica indicada fazia parte do receituário utilizado pela medicina da época para o tratamento da lepra, que não fugia à diversidade das práticas de cura comuns no século XIX, tais como o uso de sudoríficos, diuréticos, catárticos, visicatórios e mercuriais. No Hospital dos Lázaros no Rio de Janeiro, um dos principais centros de etiologia e tratamento da enfermidade, era usada toda sorte de terapias (Cabral, 2006b, p.42-43). Ficaram conhecidos os testes produzidos com "os sucos de plantas e raízes, como a coroa-de-frade (*melocactus bahiensis*), pinhão-manso (*jatropha curcas*), figueira-brava (*fícus*), mandioca-amarga (*manihot*

*utilíssima*) e o inhame ingerido como alimento" (Lima, 2021, p.48). A inexistência de uma terapêutica específica para a lepra e outras doenças no período explica-se, em grande medida, por fatores multicausais então associados à sua etiologia. Em vista disso, acreditava-se que um dos principais papéis do médico era contribuir para reestabelecer o equilíbrio natural do organismo (Rosenberg, 1977; Cabral, 2006a, p.42).

## Rotina, trabalho e disciplina

Outro aspecto a ser notado no documento diz respeito à rotina a ser adotada pelos enfermos. Ottoni (8 jan. 1855, fl.20) propunha que o lazareto fosse "afastado de moradores e exalações nocivas". Logo que chegassem, os doentes deveriam ser "divididos em classes, segundo o estado da moléstia e outras considerações". As acomodações, ou "celas", seriam divididas em duplas de internos, tendo cerca de três metros quadrados e janelas somente para os corredores. Visitas seriam permitidas somente em "horas determinadas e com a permissão do Médico". Para o bom funcionamento, o hospital deveria contar com corpo de funcionários. O mordomo, funcionário a quem Ottoni dava protagonismo, seria o principal responsável pelos doentes, figurando como uma espécie de "chefe de família e deverá governá-la com caridade e amor de pai. ... Compete-lhe suprir do necessário e prestar contas ao Governo". Foi a única função que Ottoni destacou como desejável que vivesse internamente no lazareto. O boticário cuidaria da "botica e jardim das plantas medicinais". Ao médico competiria "a direção e prescrição do tratamento dos morféticos ... colher observações regulares para formar anualmente a estatística dos doentes que tratar e o relatório dos métodos terapêuticos", além de zelar pela regularidade do "trabalho e recreio dos doentes". Solicitava ainda uma secretaria, sem funcionários e funções definidas, e um capelão para a capela do lazareto, "para os doentes exercerem nela o culto Divino" (Ottoni, 8 jan. 1855, fl.20).

Por último, haveria número indefinido de enfermeiros. Não por menos, além do auxílio aos médicos, esperava-se dos enfermeiros a observância da rotina dos adoecidos. Espaços e trabalhos seriam compartilhados, mas com divisões de acordo com os sexos. Haveria salões únicos para refeitório e para enfermaria, sendo ambos separados "em duas partes iguais. Uma para homens e outra para mulheres". O mesmo serviria para o terreno dos jardins, pomares, plantações, hortas e espaço de recreação dos doentes (Ottoni, 8 jan. 1855, fl.6).

O trabalho era incentivado, mas deveria ser "proporcionado às forças de cada um". Além dos já mencionados plantios de hortas, pomares e jardins, o médico recomendava que as mulheres em menor estágio de adoecimento poderiam se ocupar "na costura e noutros misteres domésticos e os homens no asseio do estabelecimento". Destacava que se deveria ter "mais em vista a cura dos doentes do que o lucro do estabelecimento". De modo complementar à terapêutica, além de promover o "equilíbrio das funções do organismo", tal preceito retirava os indivíduos do ócio e os afastava de seus vícios, como a libido, traço predominante nos morféticos, segundo o discurso médico da época. Parafraseando Ovídio, Ottoni dizia que "se tirares a ociosidade do mundo, quebrarás as flechas do amor" (Ottoni, 8 jan. 1855, fl.8-9).

Michel Foucault (1999) aponta que a exclusão do leproso em espaço determinado ganhou força no século XIX. Os portadores da doença eram tirados do convívio social e instalados em espaços de observação e disciplina não só do tratamento da doença, mas do comportamento do doente. Pode-se traçar um paralelo entre o estabelecimento da rotina e da disciplina no lazareto e o funcionamento das instituições totais. De acordo com a definição de Erving Goffman (2007, p.11), uma instituição total pode ser definida como um "local de residência e trabalho onde grande número de indivíduos com situação semelhante, separados da sociedade mais ampla por considerável período de tempo, levam urna vida fechada e formalmente administrada".

Assim, isolar morféticos em lazaretos, como proposto por Ottoni, não seria, necessariamente, prática de clausura ou encarceramento, mas, sim, de os estabelecer em local de rotina médica e social bem circunscrita, "onde os menores movimentos são controlados, onde os acontecimentos são registrados" (Foucault, 1999, p.222-223).

Ainda que o objetivo do trabalho declarado aos enfermos fosse o salutar, seria possível que, por meio dele, se abatessem custos de internação. Sobre esse aspecto, o planejamento de Ottoni demonstra alguns detalhamentos. Aos doentes pagantes, as internações seriam custeadas na forma de pensionato, calculando-se em diárias. Aos que pudessem trabalhar, o mordomo seria incumbido de registrar em livro "os lucros que tiver ganho cada doente" (Ottoni, 8 jan. 1855, fl.21). Metade dos ganhos seria usada para tratamento, e a outra metade seria direito do doente quando saísse do estabelecimento. Haveria ainda os internos gratuitos, isto é, escravizados, indigentes e mais pessoas que não pudessem arcar com o tratamento (fl.21).

Era desejável que todo cotidiano de tratamento médico, rotina de trabalho e vigilância sexual ocorresse sem que fosse necessário "de modo algum usar de qualquer meio violento". Todavia, também era previsto que médico e mordomo pudessem "determinar os meios repreensivos e coercivos" que fossem necessários "impor aos doentes quando sejam indispensáveis para obrigá-los à obediência". Segundo Ottoni (8 jan. 1855, fl.20), as punições disciplinares do Hospício Pedro II seriam adaptáveis ao lazareto, que poderiam chegar a: "1º Privação de visitas, passeios e quaisquer outros recreios. 2º A diminuição de alimentos dentro dos limites prescritos pelo Médico. 3º A reclusão solitária com a cama e os alimentos que o Médico prescrever, não excedendo a 2 dias de cada vez".

As sugestões disciplinares de Ottoni não previam expulsão ou qualquer tipo de exclusão do hospital. O documento, aliás, demonstra que saídas de qualquer natureza só seriam permitidas mediante a obtenção de "um curativo completo", com exceção dos "pensionistas, os quais poderão obter alta seja qual for seu estado, satisfeitas as despesas vencidas" (Ottoni, 8 jan. 1855, fl.22).

## Algumas questões médico-científicas

O parecer fornece ainda subsídios para compreender alguns aspectos do debate científico em torno da lepra na primeira metade do século XIX. Esse período foi marcado por esforços científicos para estabelecer uma nosologia específica acerca da doença, de acordo com os parâmetros da anatomoclínica e climatologia médica (Cabral, 2013, 2006a). As duas especialidades constituíam os fundamentos da autoridade científica na medicina do período (Edler, 2003).

Com relação à transmissibilidade, Ernesto Ottoni (8 jan. 1855, fl.9) também compartilhava da ideia de que a doença era hereditária e que tal predisposição poderia ser combatida pela educação, como meio de não perpetuar a “raça destes infelizes, nunca rompendo laços de família já formados, mas evitando que novos se formem”. Nesse sentido, conforme mencionado acima, suas ideias concordavam com as proposições que objetivavam evitar o ato sexual entre os doentes e destes com as pessoas sãs, visando, assim, conter a “multiplicação dos morféticos” (Cabral, 2006b, p.40). A tese que atribuía a lepra à hereditariedade se deve aos médicos noruegueses Daniel Cornelius Danielssen (1815-1894) e Carl Wilhelm Boeck (1808-1875), em 1848. Segundo Jaime Benchimol e Magali Romero Sá (2004, p.27), embora “não descartassem a relação com condições desregradas e anti-higiênicas de vida, e com o meio ambiente insalubre, propugnada pelo paradigma neo-hipocrático para muitas outras doenças, Danielssen e Boeck sustentaram que a lepra era uma doença essencialmente hereditária”.

Desse modo, nota-se que, no parecer, o médico indicava estar a par da literatura sobre a doença no período. Entre os médicos mencionados em seu relatório, realça o douto João Maurício Fraive, que também refutava o caráter contagioso da moléstia. Esse médico publicou duas memórias, em 1845, sobre a enfermidade, apoiando-se na descrição dos casos clínicos e em autópsias. Entre outros aspectos assinalados por ele, com base no paradigma anatomoclínico, supunha, pela observação clínica da anestesia, que se tratava de afecção do sistema nervoso (Cabral, 2006a, p.37).

Apesar de algumas menções a outros autores pesquisadores da lepra e alguns leprosários no Brasil, Ernesto Ottoni demonstra ter em Fraive sua principal referência e, particularmente, a experiência do médico francês com o lazareto de Caldas Novas, na província de Goiás. Para defender o isolamento dos morféticos, e que, segundo ele, “o próprio interesse dos doentes os convida para esta vida retirada”, o médico cita literalmente o relato daquele médico sobre os bons resultados obtidos em Goiás, ao dizer que os adoecidos

> formam uma irmandade, que o infortúnio comum torna mais íntima, lá vi mais caridade do que entre nós, vivem ali em sossego, suciam, parolam, conseguem de quando em quando divertir-se, que não lhes é lícito no mundo, que, apesar do instinto da humanidade, os aborrece. A essa influência moral, a salubridade do lugar, ao extremoso asseio entretido pela abundância das águas, se deve, portanto, atribuir o melhoramento de alguns sintomas da moléstia, e mesmo de estado geral da saúde experimentada por alguns doentes (Ottoni, 8 jan. 1855, fl.15-16).

Em contraste, no parágrafo seguinte da correspondência, realça o mau exemplo: “O estado desgraçado a que se acham reduzidos estes infelizes nas Províncias de Minas e São Paulo, dominadas de ordinário do preconceito da incurabilidade do mal”. Nesses locais, segundo Ottoni (8 jan. 1855, fl.16), viviam os adoecidos sem acolhimento médico e humanitário, dispersos pelas ruas, relegados à miséria e, “sem encontrar lenitivo algum a seus males, entregam-se a todos os horrores da sua sorte, se não chegam mesmo atentar contra seus dias”.

O argumento mostra-se elucidativo no sentido de que os leprosários serviriam de estratégia de luta contra a lepra, mas também contra todos os impactos que os acometidos pela enfermidade produziam na sociedade. Na concepção da época, os portadores da doença eram associados aos vícios da mendicância, à vadiagem, à promiscuidade e à

loucura. Além disso, havia a percepção estigmatizada da doença, pelas marcas que deixava nos corpos dos enfermos. Tudo isso deveria ser afastado das cidades, onde os dirigentes mostravam-se cada vez mais incutidos do ideal de higiene coletiva. Ao mesmo passo, aos acometidos pelos sobreditos males cabia a correção dos desvios, preferencialmente isolados em hospitais, asilos, penitenciárias e, no caso dos morféticos, em lazaretos (Foucault, 1999, p.223). Tratamento médico, observação à doença, respeito ao doente, acolhimento à pobreza, rotinas salutares, lazer, trabalho e disciplina são os aspectos mais enfatizados no relatório de Ernesto Ottoni. Seu projeto serviu de base para uma planta do edifício. Embora a Assembleia Provincial tivesse aprovado recursos para o empreendimento, o projeto não foi adiante, pois o orçamento excedia os recursos da província. O presidente que assumiu o lugar do conselheiro Saraiva, doutor José Joaquim, opôs-se ao projeto, pois o lazareto não comportaria todos os doentes da província e não seria fácil isolar os doentes (Maurano, 1950, p.85). Tal fato, a nosso ver, não invalida o significado do parecer de Ottoni, o qual pode ser visto como uma fonte importante para a reconstituição da história da lepra em São Paulo, as propostas de assistência aos enfermos naquele contexto, bem como a forma pela qual a doença era compreendida.

Fontes documentais, como o relatório de Ernesto Ottoni, são de suma importância para a historiografia que objetiva analisar doenças, medicina e ciências no Brasil do século XIX. Além de permitir conhecer a atuação de Ottoni no combate e construção do conhecimento sobre a lepra no Brasil oitocentista, a transcrição detalhada do relatório realça novas perspectivas sobre a trajetória do médico, destacando-se entre algumas das questões aqui apresentadas: sua visão sobre a relação entre o doente e a doença, a importância dada ao isolamento para o tratamento, além de seus alinhamentos e ressalvas com o que outros homens de ciência propunham para o tratamento da enfermidade e diminuição de sua propagação. Por fim, ressalta-se que trabalhos como este são possibilitados em razão do esforço de instituições privadas e públicas, a exemplo do Arquivo do Estado de São Paulo, que digitalizam acervos e disponibilizam fontes originais, contribuindo para maior alcance, diversificação e conhecimento de temas e personagens da historiografia.

## Sobre a transcrição

O tipo de transcrição escolhido foi a modernizada, isto é, que atualiza a ortografia das palavras apresentadas em documento, respeitando-se somente a gramática e a pontuação conforme o original (Arquivo Nacional, 1993; Acioli, 2003). Ainda que a escrita do documento se mostre relativamente próxima à ortografia contemporânea, escolheu-se esse tipo de transcrição com a finalidade de aumentar o alcance explicativo da fonte. Reforça-se, de todo modo, que a atualização da grafia não traz prejuízo à compreensão do sentido do texto, à intencionalidade dos autores, tampouco sobre seu contexto de produção. Ao todo, são 23 fólios, numerados sequencialmente, a partir do fólio 2, registrados em 23 imagens que reproduzem o conteúdo original do documento em sua totalidade. Não consta qualquer indicação de que o documento se trate de cópia, traslado ou demais tipos de reprodução que possam incidir em edição ou prejuízo de sentido do proposto pelos autores. Da mesma forma, não há marcas ou corrosões documentais que tornem qualquer parte da leitura inconclusiva.

## NOTAS

[1] No século XIX, a morfeia era uma das formas de designar a lepra, assim como o mal de Lázaro, a elefantíase dos gregos. Sobre as discussões em torno das terminologias utilizadas para se referir à doença no período ver, entre outros, o estudo de Cabral (2006a).

[2] A colonização do vale do Mucuri, onde foi fundada a freguesia da Filadélfia, envolvia constituição de núcleos urbanos, de portos e incentivo à imigração estrangeira. Além dos colonos que afluíam para a região, a partir de 1856, mediante acordos com o governo central, vieram imigrantes alemães, belgas, portugueses, franceses e chineses (Duarte, 2002).

[3] O relatório foi publicado também no *Correio Paulistano*, periódico utilizado pelos presidentes da província para publicar relatórios e outros documentos atinentes à administração pública.

[4] A construção do edifício data de 1802. Maurano (1950, p.102) afirma que o hospital só começou a funcionar em 1825, e Maciel e Ferreira (2014, p.22) datam a criação do estabelecimento em 1805.

## Transcrição

Emite o parecer referente às necessidades de se construir um local para os doentes. Situa a Chácara de Sant'Anna como local perfeito para tal edificação por sua localização e salienta a necessidade de se cumprir as leis de higiene, salubridade e beleza na arquitetura.

2

<2>

O. 902
C. 107
P. 06
D. 001

Ilustríssimo Excelentíssimo Senhor

Tendo sido nomeado por Vossa Excelência para fazer parte da comissão incumbida de emitir sua opinião relativamente ao disposto no artigo 33 do Orçamento Provincial, que autoriza a Presidência à formular um regulamento policial e sanitário para os morféticos mendigos, em minha franca opinião entendo, que para se poder observar bem essa disposição da lei, cumpre fundar antes em estabelecimento regular, onde estes infelizes encontrem o sustento necessário e em tratamento metódico: seria talvez mais conveniente a fundação de diferentes Lazaretos nos lugares, em que a morfeia grassa endemicamente; mas estabelecimentos destes só oferecem garantia de utilidade e conservação, sendo fundador em lugares acessíveis a todos os recursos debaixo das vistas, e imediata proteção do Governo: assim pois farei algemas observações relativas à fundação de um estabelecimento tão altamente reclamado pelas necessidades públicas nas proximi-

<3>

dades desta Capital.

Nenhum lugar encontro aqui mais adequado para este fim de que a Fazenda de Santa Ana, situada ao norte da Cidade, em distância de meia légua pouco mais ou menos, contendo um terreno elevado e seco, afastado de moradores e exalações nocivas, provido de águas potáveis, e que chegam para o asseio, gozando de uma exposição favorável aos raios do sol e os ventos, que renovam constantemente a atmosfera: com tais condições considero boa a aquisição deste lugar, não só em relação ao estabelecimento em si mesmo como em relação à Cidade e aos ventos reinantes.

Tem proporções para um estabelecimento em ponto grande; mas por ora julgo bastante em edifício, que acomode a-200-doentes de ambos os sexos. Na construção deste edifício deve-se observar a risca as leis da Higiene e da salubridade; as balizas da arquitetura e regularidade

3

<4>

dos planos devem ceder à estas considerações essenciais.

O Edifício que serve de seminário para os Educandos fica destinado para morada dos Empregados, Secretaria, Botica, quarto para roupas; dispensa, etc. A Capelinha anexa a este Edifício fica destinada para os doentes exercerem nela o culto Divino.

Para morada dos doentes se construirá uma casa assobradada com alicerces de pedra até o nível do pavimento, e paredes de tijolo com exposição – Este-Oeste; a espessura das paredes deve ser tal que nem o extremo calor, nem o frio excessivo possam penetrá-los. O renovamento do ar se fará por janelas largas rasgadas até em cima para evitar que os miasmas se acumulem na parte superior do Edifício. Ao nível do pavimento se estabelecerão ventiladores suscetíveis de abrir-se fechar-se à vontade à fim de renovar o ar da parte inferior do Edifício. As janelas

<5>

serão abertas em frente umas das outras e de fronte à porta do corredor. As enfermarias serão dispostas de maneira que durante uma parte do dia possam ser penetradas pelos raios do sol.

Observam-se perfeitamente estes preceitos higiênicos em duas ordens de celas separadas por um largo corredor com janelas nas extremidades. As celas acomodarão dois doentes cada uma para que terão pelo menos 15 palmos de largo e 18 de fundo, e uma larga janela de fronte da porta do corredor.

As paredes das celas serão ligeiras e não chegarão até o teto para deixar livre a circulação do ar. Uma sala com um único portão de entrada dividirá a enfermaria em duas partes iguais, uma para os homens, e outra para as mulheres; nesta sala haverão duas portas laterais para as respectivas enfermarias e uma de fronte da porta da entrada

4

<6>

para um corredor que vai ter a cozinha: aos lados deste corredor existirão duas salas, que servirão de refeitório, uma para os homens, outra para mulheres, parte destas salas será contida no edifício e parte em um lanço fora, no fim do qual existirá a cozinha. Os refeitórios terão janelas, e comunicação para um pátio fechado para criação de aves domésticas, e para o corredor das respectivas enfermarias: os refeitórios se comunicarão com a cozinha por meio de uma roda, que conduza a comida, e necessário. Nas extremidades do Edifício e aos lados da janela do corredor se farão duas latrinas para cada enfermaria, e uma sala de banhos. Além destes cômodos podem se fazer duas enfermarias em dois lanços paralelos ao lanço do meio, que acomodem uma parte dos doentes.

O terreno da Fazenda convenientemente cercado será destinado para plantação de hortas, jardins e pomares para passeio e recreação dos doentes: este terreno será dividido em dois, um pa=

<7>

um para ser cultivado pelos homens e outro pelas mulheres: nesta cultura deve-se respeitar os arvoredos existentes; na nova plantação deve-se ter em vista utile et dulce. O pomar constará de arvoredos e plantações, que não produzam frutos nocivos aos doentes; na horta se cultivarão as ervas frescas e antiescorbúticos, de que os doentes podem fazer uso; em mais larga escala se cultivarão as plantas de raízes tuberosas, particularmente inhame, que nos lugares, em que abunda, é considerado como preservativo da morfeia: e o que sucede nas margens do rio-doce em Minas Gerais, onde não só usam do inhame como alimento, como até cevam com ele seus porcos, entretanto mui raros casos de morfeia se observam ali.

Um jardim de plantas medicinais ficará aos cuidados do Boticário, nele se cultivarão aquelas plantas que deve ser empregadas frescas no tratamento das moléstias: em um tal

<8>

jardim deve merecer especial cuidado a cultura de plantas, que a experiência tem mostrado de eficácia no tratamento da morfeia, e com que o Senhor Doutor José da Silva, Lente de Patologia interna do Rio de Janeiro, tem enriquecido a matéria Médica Brasileira.

Os próprios doentes, que ainda estiverem no primeiro período do mal, e em estado de trabalhar, serão encarregados da cultura das hortas, jardins e pomares, tanto os homens, como as mulheres, separadamente, mas só trabalharão das 6 às 8 da manhã, e de tarde das 4 às 6 horas, em dias que não forem chuvosos nem úmidos, porque de outra forma podem aparecer incômodos funestos: durante o resto do dia as mulheres se ocuparão na costura, e noutros misteres domésticos, e os homens no asseio do estabelecimento e noutros Trabalhos. O trabalho deve ser proporcionado às forças de cada um; mas deve ser sistemático, convenientemente dirigido, tendo-se mais em vista

<9>

a cura dos doentes, que o lucro do estabelecimento.

Há de ser por certo difícil pôr em prática este sistema: mas com jeito e boas maneiras tendo se alcançará, sem que seja necessário nem convenha de modo algum usar de qualquer meio violento. Nada entretém nem distrai mais do que o trabalho.

As seguintes observações me parece que justificam a adoção deste sistema, como auxiliar da Terapêutica na cura da morfeia.

O exercício moderado e regular põe em movimento os órgãos, que não são feitos para ficar em quietação: o exercício moderado proporcionado as forças de cada um promove o equilíbrio das funções do organismo; em um indivíduo que fazer exercício moderado a digestão, a circulação a enervação tornam-se mais ativas, os movimentos respiratórios são mais frequentes, os pulmões recebem maior quantidade de ar, e também absorvem maior quantidade

<10>

de origens, a circulação capilar e a exalação cutânea participam da mesma atividade; finalmente e o organismo inteiro recebe um choque que lhe é profícuo; ora se tal é o efeito do exercício no organismo, claro está que convém muito que os morféticos se entreguem durante algumas horas do dia a um trabalho moderado e recreativo, com isso a digestão, a circulação, a enervação, a transpiração cutânea se efetuarão melhor, a sua sensibilidade não se embotará, e seus membros não cairão prontamente em completo esquecimento.

A ocupação pode até evitar as consequências da ociosidade tão prejudicada a todos, particularmente aos morféticos, em quem predomina o libido inexplebilis – considerado por muitos Médicos como um sintoma da moléstia; e que pode ser acalmado pela observação do preceito de Ovídio. Se tirares a ociosidade ao mundo, quebrarás as flechas de amor. Olia si settas periere cupidinis arcus.

Desgraçadamente ainda hoje a morfeia passa

<11>

por incurável; mas nem por isso deve-se cruzar os braços nem observar impassível seus estragos, quando aliás os anais da ciência referem algumas curas: Alibert cita alguns fatos destes devidos a Larrey, Lorre, Robinson, Valentin, e outros benfeitores da humanidade: entre nós o Senhor Doutor Silva, com plantas indígenas, e uma terapêutica sua, afirma ter curado alguns morféticos, se entre outros apresenta o fato da cura importante da Irmã do Senhor Doutor Raposo, que já se os achou no último período do mal.

Além destes fatos, se acaso refletir-se que a morfética grassará outrora com intensidade inaudita na Europa, de sorte que no tempo das Cruzadas, só no território Francês haviam 2000 Lazaretos e 19000 em toda a Europa, destinados unicamente para os morféticos, bem que alguns doentes de outras moléstias de pele se recolham a estas casas; mas que entretanto o mal limitar-se a alguns pontos, de sorte que hoje, diz em

7

<12>

Autor moderno = A Elefantíase dos Gregos é moléstia rara e pouco conhecida em França, particularmente em Paris, onde apenas se observa um ou outro caso em indivíduo, que tem trazido o germen de lugares, onde ele grassa endemicamente. Necessariamente concluise que este mal, ou foi debelado pelos princípios da Arte, ou foram ao menos obviadas as causas que o produziam. E com razão parece que se pode atribuir a sua diminuição (pois que se coincidem as épocas) aos progressos da civilização e da indústria, à propagação da luzes, no desenvolvimento de todos os ramos das ciências, e por consequência ao melhoramento dos hábitos, e dos costumes do povo, à melhor observância das leis da higiene pública e privada. Pensam sumidades Médicas do Brasil, que a morfeia também diminui sensivelmente entre nós, e atribuem, e não vê-se com razão as mesmas causas, que na Europa, e mais que tudo a cessação do tráfico de Africanos. Ora se morféticos tem sido curados, como afirmam

<13>

Autoridades respeitáveis, se este hediondo mal debaixo da suficiência de certas causas desapareceu completamente de vários lugares da Europa, onde grassava antes epidemicamente, como refere a História; se no Brasil começa a diminuir depois da cessação do tráfico de africanos, como observa o Senhor Doutor Silva e outros Médicos, cumpre que os homens da ciência envidem seus esforços contra um inimigo tão terrível, e se fazem auxiliados pelo Governo do país, talvez em pouco tempo consigam este feliz resultado essas bênçãos da posteridade, assim como são abençoados aqueles que ainda indiretamente conseguiram a extinção deste horrendo mal, de vários pontos da Europa.

Acreditam grande número de Médicos, que a morfeia não é contagiosa, o Senhor Doutor [Faivre] que teve ocasião de praticar em Goiás muitas autópsias em morféticos, exprime-se assim a respeito do contágio. = Por experiências diretas, se bem que involuntárias, ferindo-me muitas

8

<14>

vezes com esquirolas de ossos e instrumentos imbuídos de pus e sangue, obtive plena confirmação de que a moléstia não é contagiosa;; As comunicações as mais frequentes e aproximadas como as que se dão entre mulher e marido não bastam para determinar a transmissão do mal, fatos destes observei na Província de Minas e dão nesta Cidade; mal de nós se a morfeia fosse contagiosa! percorrem as ruas da cidade turmas de mendigos implorando a piedade pública, o pão quotidiano, e alguns já no último período do mal com os pés e mão trincados, cobertos de úlceras imundas e fétidas, dos morféticos que a Misericórdia trata, muitos estimulados pelo – libido inexplebilis, de que já falei confundem-se de noite com o povo que percorre as ruas, dando talvez ocasião à exposição, que teve lugar há poucos dias de esma linda criança no Hospital de lázaros, entretanto são raros os casos de morfeia nesta cidade, os morféticos que pedem esmolas, e os que

<15>

são tratados pela Misericórdia trazem em grande parte o mal de outros lugares desta Província onde grassa endemicamente.

Concordam os Autores que a morfeia se transmite pela geração dos pais aos filhos: mas nem toda a descendência destes infelizes contra a herança fatal, a predisposição pode ser combalida pela educação; todavia cumpre obviar a meio de perpetuar a raça destes infelizes, nunca rompendo laços de família já formados, mas evitando que novos se formem. O sequestro dos doentes na idade média em lazaretos, onde encontrarão todos os recursos, se deve talvez em grande parte a quase extinção do mal de muitos lugares.

O próprio interesse dos doentes os convida para esta vida retirada. O Doutor [Faivre] diz a respeito dos morféticos que se achavam reunidos nas Caldas novas de Goiás "- Eles formam uma irmandade, que o infortúnio comum torna mais íntima, lá vi mais caridade do que entre nós, vivem ali

9

<16>

em sossego, suciam, parolam, conseguem de quando em quando divertir-se, que não lhes é lícito no mundo, que, apesar do instinto da humanidade, os aborrece. A essa influência moral, a salubridade do lugar, ao extremoso asseio entretido pela abundância das águas, se deve portanto atribuir o melhoramento de alguns sintomas da moléstia, e mesmo de estado geral da saúde experimentada por alguns doentes.

Entretanto é para lastimar o estado desgraçado a que se acham reduzidos estes infelizes na Província de Minas e São Paulo, dominadas de ordinário do preconceito da incurabilidade do mal, despertados pelo horror e medo do contágio, que incutem na sociedade, sem encontrar limitivo algum a seus males, entregam-se a todos os horrores da sua sorte, se não chegam mesmo atentar contra seus dias. De que misérias não serão vítimas os infelizes, que abundam em vários pontos desta Província, e que a morfeia tem reduzido a

<17>

mendacidade? quando aqui na Capital os infelizes, que se reconhecem no atual hospital de lázaros virem em completo abandono, e morrem à míngua sem receberem o menor socorro da Medicina.

É certo que a Misericórdia dá todos os dias a cada em uma ração de alimento substancial, concede-lhes ampla liberdade, mas esta alimentação, que chegaria para sustentar um jornaleiro, é por sua natureza prejudicial e inútil para estes infelizes, particularmente quando se acham acometidos de moléstias agudas, ou quando a morfeia fazendo progresso leva uns estragos ao aparelho digestivo, produzindo fastio, e completa inaptidão para uma alimentação forte.

Consta que as queixas de suas necessidades mais urgentes não atendidas, quando ouvidas dos altos Funcionários da Irmandade; mas isto nem sempre acontece, ou seja por falta de caridade do atual mordomo, ou porque entenda que a Misericórdia não tem recursos e que já não é pequena a es=

10

<18>

mola que faz a estes desgraçados, debalde imploram muitas vezes os socorros que precisam; entretanto com a adoção de um sistema mais regular podia talvez a Misericórdia com a mesma despesa que faz hoje, evitar para o futuro a reprodução do fato ocorrido há poucos dias entre o Mordomo do lázaros e os morféticos, bradando-lhe estes junto ao leito de uma moribunda doente, que debalde pedia um caldo de galinha e marmelada em lugar de carne e feijão = Os Senhores dizem que são de Misericórdia, pois deem o que precisamos, ou então por caridade mandem-nos nos fuzilar ali no campo.

Estas considerações mostram a necessidade urgente de fundar-se nas proximidades da Capital um estabelecimento com os recursos necessários para asilo dos infelizes acometidos deste hediondo mal; nenhum lugar encontro mais adequado para este fim de que a Fazenda de Santa Ana. Fundado ali o Hospital de baixo

<19>

dos preceitos da higiene e da salubridade, deve ser dirigido por um regulamento interno que marque as atribuições dos Empregados: este regulamento será sistematicamente formulado e aperfeiçoado pelas reformas, que a experiência em certo espaço de tempo for indicando. Constará o pessoal do Estabelecimento de em Mordomo, Médico; Boticário, e um Capelão, e os enfermeiros necessários.

O Mordomo dará o chefe da família, deverá governa-la com caridade e amor de pai, e efetivamente residirá no estabelecimento; compete-lhe suprir do necessário, e prestar contas ao Governo, para o que deve ter uma escrituração regular.

O Boticário servirá de escrivão, terá cuidado na Botica, e jardins das plantas medicinais.

O Médico visitará os doentes todos os dias, e mais de uma vez se for necessário, compete-lhe a direção e prescrição do tratamento dos morféticos. Regular a ocupação, o trabalho e recreio dos doentes a horas fixas e regulares. Determinar os

<20>

meios repreensivos e coercivos, que forem precisos impor nos doentes quando sejam indispensáveis para obriga-los à obediência. Conceder prêmios quando merecerem.

Não tendo sido estudada convenientemente a Morfeia no país, cumpre ao Médico colher observações regulares para formar anualmente a estatística dos doentes, que tratar, e o relatório dos métodos terapêuticos empregados por ele em cada um dos doentes.

São adaptáveis a este estabelecimento os meios repreensivos e coercivos usados no hospício de Pedro 2º, a saber, 1º - Privação de visitas, passeios e quaisquer outros recreios. 2.º A diminuição de alimentos dentro dos limites prescritos pelo Médico. 3.º A reclusão solitária com a cama e os alimentos que o Médico prescrever, não excedendo a 2 dias de cada vez.

Os doentes que se mostrarem mais idôneos serão encarregados por acordo do Mordomo e do Médico

<21>

de dirigir os trabalhos dos jardins, hortas, pomares e outras ocupações do estabelecimento.

O Mordomo em um livro próprio assentará os lucros que tiver ganho cada doente, por um cálculo razoável em cada mês deste lucro o doente terá direito a metade quando sair curado do estabelecimento.

O regulamento interno marcará a tabela dos alimentos que os doentes podem usar.

Serão admitidas gratuitamente neste hospital as pessoas indigentes, os escravos de senhores que não possuírem mais de um, sem meios de pagar as despesas de seu tratamento e curativo. Também serão admitidos para se tratar neste estabelecimento, pagando, os doentes abastados que quiserem se tratar na companhia das pessoas acometidas do mesmo mal, que não as expelem nem as aborrecem. Os escravos de senhores que podem pagar o seu tratamento e curativo.

O regulamento interno marcará quanto cada

12

<22>

pensionista pagará por dia.

Serão admitidos no estabelecimento aqueles que provarem o seu estado de indigência com atestado de Cura e da Polícia.

Os pensionistas não serão admitidos sem que apresentem ao Mordomo em escrito assinado por pessoa notoriamente abonada, que afiance o pagamento mensal das quotas diárias.

Os doentes na ocasião da entrada serão divididos em classes segundo o estado da moléstia e outras considerações.

Os morféticos não poderão sair do Hospital sem obterem um curativo completo, exceto os pensionistas, os quais poderão obter alta seja qual for seu estado, satisfeitas as despesas vencidas.

Nenhum doente poderá obter alta senão nos dois casos acima, não poderão receber visita, senão à horas determinadas e com a permissão do Médico. Ficarão completamente sujeitos à determinação do regulamento interno.

<23>

O Governo convidará a todos os morféticos que se acham espalhados pela Província para virem se utilizar deste pio estabelecimento, do qual o bom tratamento e regularidade sejam os mais fortes incentivos, não só de atrair mendigos como até os doentes abastados, e se em compensação não obtiverem a cura do seu mal ao menos viverão aqui mais felizes.

São estas as observações, que me ocorrerão, prescindi d'outras considerações relativamente a morfeia e à suas causas, para evitar a prolixidade; a magnitude porém do objeto, e o desejo de concorrer com meu fraco contingente para a fundação de um estabelecimento tão útil, me fez talvez sair dos limites que me eram prescritos neste parecer. Releve Vossa Excelência a imperfeição do trabalho pela boa vontade que tinha de coadjuvar a Vossa Excelência nos esforços e boas intenções que Vossa Excelência tem mostrado em favor da humanidade; pois pela minha parte antes

<24>

quero prestar serviços destes a meu país do que outros nos versáteis campos da Política.

Deus Guarde a Vossa Excelência – São Paulo 8 de janeiros de 1855.
Ilustríssimo Excelentíssimo Senhor Doutor José Antônio Saraiva
Presidente desta Província

Doutor Ernesto Benedicto Ottoni
